# Circadian rhythm of PERIOD2::LUCIFERASE expression in the trigeminal ganglion of mice

**DOI:** 10.3389/fnins.2023.1142785

**Published:** 2023-03-28

**Authors:** Yukie Shirakawa, Sachi N. Ohno, Kanae A. Yamagata, Eriko Kuramoto, Yoshiaki Oda, Takahiro J. Nakamura, Wataru Nakamura, Mitsutaka Sugimura

**Affiliations:** ^1^Department of Dental Anesthesiology, Graduate School of Medical and Dental Sciences, Kagoshima University, Kagoshima, Japan; ^2^Department of Oral Anatomy and Cell Biology, Graduate School of Medical and Dental Sciences, Kagoshima University, Kagoshima, Japan; ^3^Department of Oral Chrono-Physiology, Graduate School of Biomedical Sciences, Nagasaki University, Nagasaki, Japan; ^4^Laboratory of Animal Physiology, School of Agriculture, Meiji University, Kawasaki, Kanagawa, Japan

**Keywords:** Circadian rhythm, Suprachiasmatic nucleus, Trigeminal ganglion, PER2, Cryptochrome

## Abstract

**Introduction:**

The trigeminal nerve conveys delicate sensations such as warmth, pain, and tactile pressure in the oral and facial regions, and most trigeminal afferent cell bodies are located in the trigeminal ganglion. Our previous study has shown that sensations in trigeminal nerve innervated areas, specifically in the maxillofacial region, exhibit diurnal variation and that sensitivity changes time-dependently. In this study, we aimed to clarify the rhythm of expression of clock gene in the trigeminal ganglion of mice to elucidate the mechanism of circadian regulation in the same area.

**Methods:**

Immunohistochemistry examined the expression of the PER2 protein in the suprachiasmatic nucleus and trigeminal ganglion of wild-type mice. To measure gene expression as bioluminescence, PERIOD2::LUCIFERASE knock-in (PER2::LUC) mice were used. Unilateral trigeminal ganglion and brain sections including the suprachiasmatic nucleus were incubated *ex vivo*. Bioluminescence levels were then measured using a highly sensitive photodetector. The same experiments were then conducted with *Cry1* gene-deficient (*Cry1^−/−^*) or *Cry2* gene-deficient (*Cry2^−/−^*) mice.

**Results:**

In the trigeminal ganglion, immunohistochemistry localized PER2 protein expression within the neuronal cell body. Mouse trigeminal ganglion *ex vivo* tissues showed distinct circadian oscillations in PER2::LUC levels in all genotypes, wild-type, *Cry1^−/−^*, and *Cry2^−/−^*. The period was shorter in the trigeminal ganglion than in the suprachiasmatic nucleus; it was shorter in *Cry1^−/−^* and longer in *Cry2^−/−^* mice than in the wild-type mice.

**Conclusion:**

The expression of *Per2* in neurons of the trigeminal ganglion in *ex vivo* culture and the oscillation in a distinct circadian rhythm suggests that the trigeminal ganglion is responsible for the relay of sensory inputs and temporal gating through autonomous circadian oscillations.

## 1. Introduction

In mammals, circadian oscillations are inherent in various organs and tissues, affecting sleep/wakefulness, hormone secretion, and other factors (Hoekstra et al., 2021; Li et al., 2021). Disruption of the suprachiasmatic nucleus (SCN) causes loss of behavioral rhythms, and other disturbances in physiological functions throughout the body, and demonstrates that the central clock is localized in the SCN (Nagai et al., 1978). Transgenic animals expressing the luciferase reporter for clock gene expression have been developed, and circadian oscillations were observed in their cultured muscle and peripheral organs (e.g., lungs and liver), confirming the existence of both central and peripheral clocks (Yamazaki et al., 2000; Yoo et al., 2004). The mammalian circadian system is an oscillatory hierarchical structure consisting of a central clock in the SCN and a group of peripheral clocks in the other tissues. The central clock entrains each peripheral clock rhythmically to the light cycle, regulating physiological functions throughout the body. In contrast, the peripheral clock is thought to regulate the rhythms of transcription of a group of genes important for the physiological phenomena in each tissue, thereby tuning this tissue to optimal temporal conditions, i.e., “to do the right things at the right time” (Aschoff, 1965). Modern society has created a phase shift between the environment and the body’s internal and peripheral clocks, and various diseases have been attributed to this change. When the body clock is disrupted by working at night or staying up late, the risk of sleep disorders (Liu et al., 2022), high blood pressure (Costello and Gumz, 2021), and cancer increases (Pariollaud and Lamia, 2020). Therefore, synchronizing the internal body clock with its environment is important.

In this study, we focus on the trigeminal ganglion, where the cell bodies of most trigeminal afferents are aggregated, as one possible peripheral clock. The trigeminal nerve conveys delicate sensations such as warmth, pain, and tactile pressure in the oral and facial regions and is the largest of the cranial nerves. The trigeminal ganglion consists of the cell bodies of afferent nerves not only of the periodontal ligament but also of the masticatory muscles and temporomandibular joint (Zhang et al., 1991; Casatti et al., 1999). In rats, trigeminal cell bodies are largely divided into large type A and small type B cells, based on structural and neurochemical differences (Kai-Kai, 1989). The cell type correlates with function, for, e.g., type A cells are associated with afferent nerves in the pulp (Sugimoto and Takemura, 1993). In addition, ganglion cells are surrounded by fibers such as noradrenergic sympathetic axons, vasoactive-intestinal-polypeptide-positive parasympathetic axons, serotonergic axons, and various peptidergic axons, such as substance P, galanin, calcitonin gene-related peptide, cholecystokinin, and nitric oxide synthase (Lazarov, 2002). In the clinic, the trigeminal-innervated area is where complaints of glossodynia, hypersensitivity, and insensitivity are localized, even in the absence of an organic oral cavity abnormality (Scala et al., 2003). Recently, we described a diurnal variation in pain in the trigeminal-innervated area in mice, with greater sensitivity to pain during the dark than during the light period; this may be due in part to diurnal variation in TRPA1 mRNA expression in the trigeminal ganglia (Niiro et al., 2021). However, the rhythm of the trigeminal ganglion itself, which produces the day-night difference, is not known. Therefore, this study aimed to examine the circadian rhythm of clock gene expression in the mouse trigeminal ganglion and to clarify the role of the *cryptochrome* (*Cry*) gene, which is known to act as a key component among clock genes in mammals.

## 2. Materials and methods

### 2.1. Ethics statement

All experiments with animals were conducted according to the National Institutes of Health Guide for the Care and Use of Laboratory Animals. This study was performed in accordance with protocols approved by the Animal Care and Use Committee of Kagoshima University (permission #D21019, D22002) and the Animal Care and Use Committee at Nagasaki University (permission #1809181479).

### 2.2. Animals

This experiment used male C57BL/6 J mice (Japan SLC, Shizuoka Japan), heterozygous PERIOD2::LUCIFERASE knock-in (PER2::LUC) mice (Yoo et al., 2004), and *Cry1* gene-deficient (*Cry1^−/−^*) and *Cry2* gene-deficient (*Cry2^−/−^*) mice (Vitaterna et al., 1999). Animals were maintained in a 12 h light/dark cycle (light intensity; 80–100 lux at cage level) for at least two weeks, with *ad libitum* access to food and water in a quiet, temperature-controlled (24–25°C) room. The light-on time was defined as Zeitgeber time (ZT) 0, and the light-off time was defined as ZT12.

### 2.3. Immunohistochemistry

Mice were anesthetized at ZT8 with an intraperitoneal injection of sodium pentobarbital (60 mg/kg), perfused intracardially with 10 mL of 10 mM sodium phosphate (pH 7.4) buffered 0.9% saline solution (PBS), and followed with 50 mL of 3% formaldehyde, 75%-saturated picric acid, and 0.1 M Na_2_HPO_4_ (adjusted to pH 7.0 with NaOH). The brains and trigeminal ganglia were removed and the trigeminal ganglia were gelatin-embedded. They were post-fixed overnight at 4°C in the same fixative and then cryoprotected in 30% sucrose in PBS (pH 7.4). The brains and the trigeminal ganglia were sliced into 40-μm-thick coronal sections and horizontal sections, respectively on a freezing microtome; the sections were sequentially stored in PBS. Every three sections were analyzed by further immunohistochemistry, and all subsequent studies were performed at room temperature (24–26°C). The sections were incubated overnight with a rabbit antibody against PER2 (1:1,000 for the brain, 1:100 for the trigeminal ganglion; PER21-A; Alpha Diagnostics, San Antonio, TX, United States) in PBS containing 1% donkey serum (AB237258; Jackson Immuno Research Laboratories, Inc., Bar Harbor, ME, United States), 0.3% Triton X-100 (PBS-X), 0.12% lambda-carrageenan, and 0.02% sodium azide (PBS-XCD). After washing in PBS-X, the sections were incubated for 4 h in PBS-XCD with donkey anti-rabbit IgG H&L (Alexa Fluor 594) (1:500; ab150068; Abcam, Cambridge, United Kingdom), NeuroTrace 500/525 Green Fluorescent Nissl Stain (1:500; N21480; Life technologies, Carlsbad, CA, United States), and 4′,6-diamino-2-phenylindole (1µg/ml; DAPI). After washing the sections thoroughly in PBS-X, they were mounted on APS-coated slide glasses (APS-01; Matsunami Glass Ind, Osaka, Japan), air dried, and cover slipped with 90% (v/v) glycerol and 2.5% (w/v) triethylenediamine (antifading agent) in 20 mM Tris-HCl (pH 7.6). The sections were observed under a confocal laser-scanning microscope (LSM 900; Zeiss, Oberkochen, Germany) with appropriate filter sets for Alexa Fluor 594 (excitation 594 nm, emission 630–800 nm), NeuroTrace 500/525 Green (excitation 450–490 nm, emission 505–530 nm), and DAPI (excitation 405 nm, emission 414–515 nm).

### 2.4. Bioluminescence imaging of the trigeminal ganglion

For real-time bioluminescence imaging, male 17- to 28-week-old wild-type (WT) mice (*n* = 4) with the heterozygous PER2::LUC gene were sacrificed at ZT 8. The trigeminal ganglion was rapidly removed and placed onto MilliCell Culture Plate Inserts (PICM ORG 50; MILLIPORE, Billerica, MA, United States) in a 35-mm glass-based dish (3970–035; IWAKI, Tokyo, Japan) containing 1.8 mL recording medium composed of Dulbecco’s modified Eagle medium (12100046; Thermo Fisher Scientific, Waltham, MA, United States) supplemented with 10 mM HEPES, 7.5% sodium bicarbonate solution, 25 μg/mL streptomycin, 25 units/mL penicillin, 5% B27 supplement (17,504,001; Thermo Fisher Scientific), and 0.1 mM luciferin (126–05111; Fujifilm Wako Pure Chemical Corporation, Osaka, Japan). The cap of the dish was sealed using silicone grease. Bioluminescence images of the trigeminal ganglion were acquired using an EM-CCD camera (iXon DU-897E; Andor Technology, Belfast, United Kingdom; frame rate: 30 min/frame; exposure time: 1790 s; EM gain: 1000) in a light-tight 36.0°C environmental chamber attached to an inverted microscope (LV200; OLYMPUS, Tokyo, Japan) with a MPLN dry objective lens (5×, 0.1 NA) (OLYMPUS). Bioluminescence images were captured for 7 days. A median filter of three frames was applied offline to reduce noise. To confirm the cellular structure, trigeminal ganglia were sliced into thin 50-μm sections and stained with 0.2% cresyl violet for Nissl staining.

### 2.5. Bioluminescence measurement in the trigeminal ganglion

For real-time bioluminescence monitoring, male 9- to 22-week-old wild-type, *Cry1^−/−^*, and *Cry2^−/−^* (*n* = 8 per genotype) heterozygous PER2::LUC knock-in mice were sacrificed at ZT 6. The brains were removed, immersed in ice-cold Hank’s buffered saline solution, and cut into 300-μm-thick sections using a D.S.K. Super Microslicer ZERO 1 (Dosaka EM Co., Ltd., Kyoto, Japan). Brain slices containing the SCN or trigeminal ganglia were placed onto MilliCell Culture Plate Inserts in a 35-mm plastic dish (1000–035; IWAKI) containing 1.2 mL recording medium as previously described. The cap of the dish was sealed using silicone grease. The SCN slices and the trigeminal ganglia were placed in a dark incubator at 36.0°C and observed using a photomultiplier tube (PMT; H9319-11; Hamamatsu Photonics, Shizuoka, Japan). The bioluminescence was counted continuously every minute. Bioluminescence data were detrended by subtracting the 24 h moving average from the raw data and smoothed by the 3 h moving average method for analysis. The first peak of the circadian rhythm was determined as the highest point in the smoothed data between 12 and 36 h after the start of the cell culture process. The period of PER2::LUC activity was calculated as the average of the difference in peak times, and the highest point of the smoothed bioluminescence data across 4 days. For statistical analysis, we used Welch’s two-sample *t*-test for comparison of the periods and peak phases in the SCN and the trigeminal ganglion. The periods and peak phases of PER2::LUC between wild-type and *Cry1^−/−^* and *Cry2^−/−^* mice were compared using one-way analysis of variance with Bonferroni’s post-hoc analysis. Results are indicated as mean ± SE, and statistical significance is defined as *p*-values <0.05.

## 3. Results

### 3.1. Immunohistochemistry of the trigeminal ganglion

**Figure 1:**
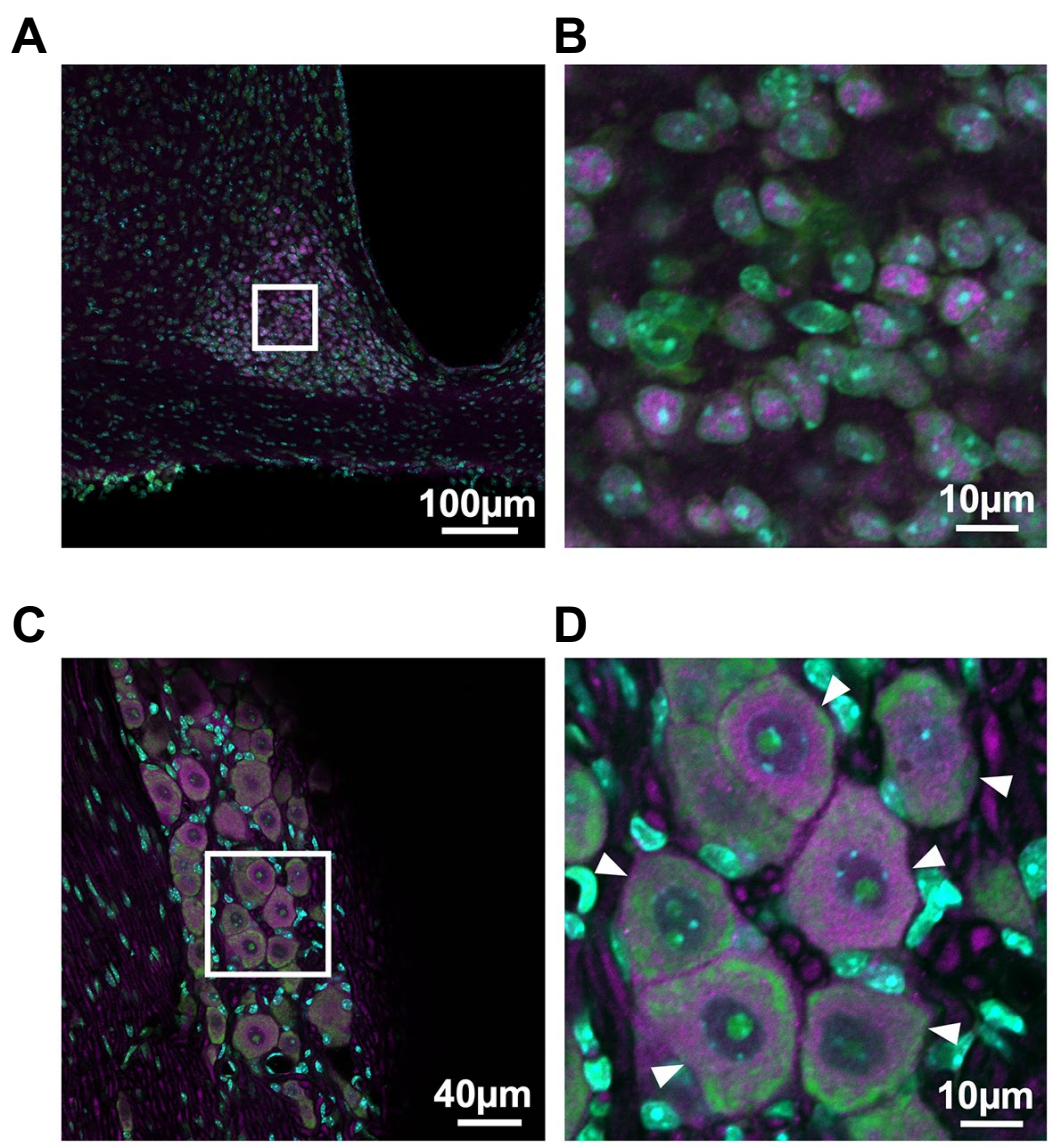
**PER2 expressions in the suprachiasmatic nucleus and the trigeminal ganglion of wild-type mice**. Representative images of immunohistochemistry of PER2-immunopositive cells in the suprachiasmatic nucleus (SCN; **A, B**) and the trigeminal ganglion (TG; **C, D**) at ZT8. Scale bar A = 100 μm, B and D = 10 μm, C = 40 μm. Green, NeuroTrace Green; cyan, DAPI; magenta, PER2. Arrowheads, neuronal cell bodies in the trigeminal ganglion.

PER2 expression in the SCN and the trigeminal ganglion was examined using immunohistochemistry to determine which cells in the trigeminal ganglion express PER2. Neurons in the trigeminal ganglion were larger than those in the SCN, and satellite cells were present around neuronal cell bodies of the trigeminal ganglion. PER2-immunopositive cells coincided with neuronal locations in both the SCN and trigeminal ganglion. In the trigeminal ganglion, PER2 was most strongly expressed in the cytoplasm of large neurons, with little expression in satellite cells. PER2 was also expressed in fibers surrounding the neuronal cell bodies (Figure 1).

### 3.2. Diurnal variation in PER2 expression in mouse trigeminal ganglion

**Figure 2:**
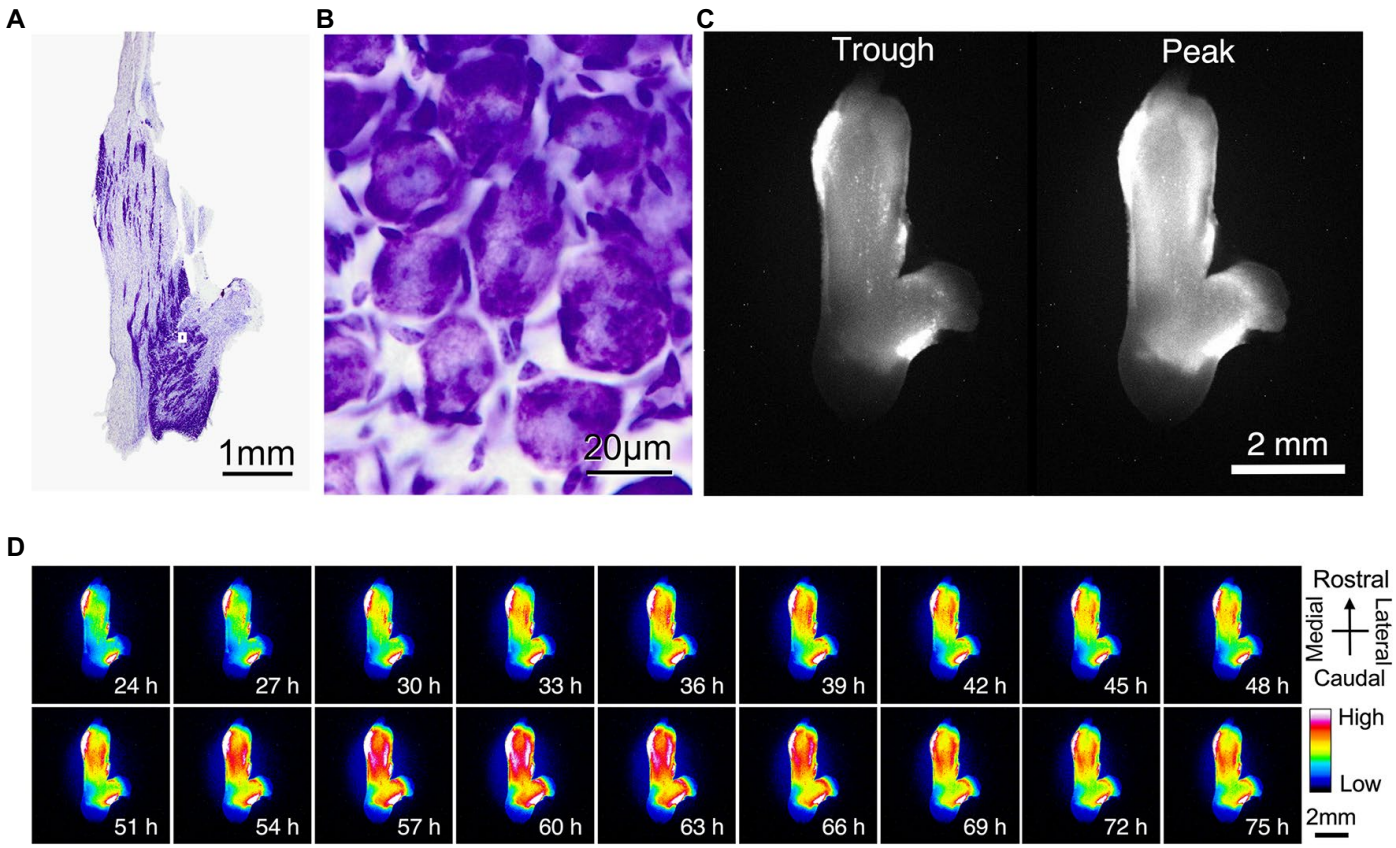
**Localization of PER2::LUC expression in the trigeminal ganglion**. **(A)** Nissl stain showing the anatomic organization of the mouse trigeminal ganglion. **(B)** Nissl stain of the mouse trigeminal ganglion at high magnification. **A, B** are the same sample. **(C)** Trough and peak of PER2 bioluminescence rhythm. **(D)** Representative images of dissected wild-type trigeminal ganglion in the dorsal view. Images are lined every 3 h for 2 days. Pseudo colors indicate bioluminescent intensities. Scale bar, 2 mm. **C, D** are the same sample.

We used bioluminescence images to determine whether PER2 expression in the trigeminal ganglion varied according to circadian rhythms. Nissl staining of the trigeminal ganglion revealed that large neurons were localized and clustered. The clusters of striated neurons could be seen between the axon fibers (Figure 2A). The neurons were surrounded by satellite cells and were located between fibers (Figure 2B). Bioluminescence imaging showed that the circadian oscillations of PER2::LUC expression are present throughout the trigeminal ganglion (Figure 2C,D).

### 3.3. Circadian rhythm in the *ex vivo* trigeminal ganglion

**Figure 3:**
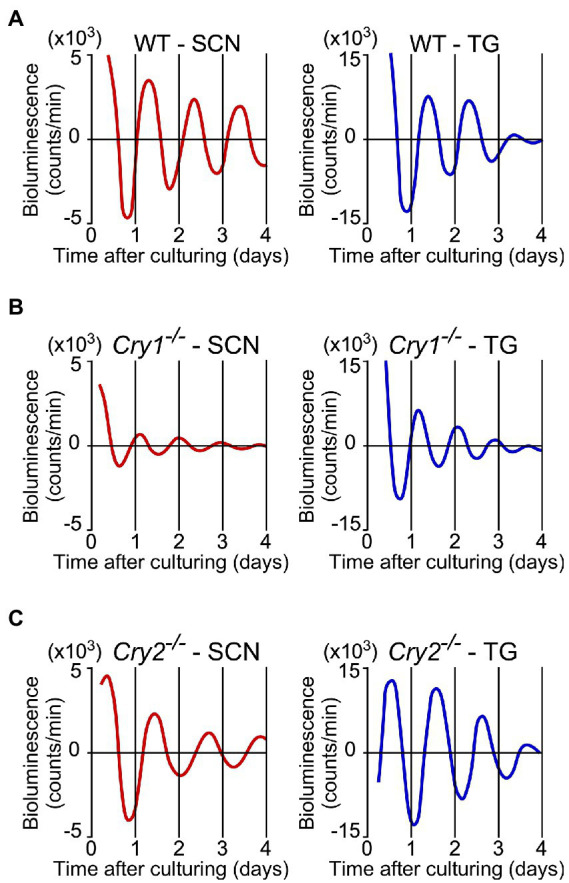
**Circadian rhythms of PER2::LUC expression in the suprachiasmatic nucleus and the trigeminal ganglion of wild-type, *Cry1^−/−^*, and *Cry2^−/−^* mice**. Representative bioluminescence rhythms of PER2::LUC expression in the suprachiasmatic nucleus (SCN; left) and the trigeminal ganglion (TG; right) of wild-type (WT; **A**), *Cry1^−/−^* **(B)**, and *Cry2^−/−^* **(C)** mice. The *x*-axis is the days from the time when the tissues were cultured. The counts of serial de-trended bioluminescence for 4 days are plotted.

**Figure 4:**
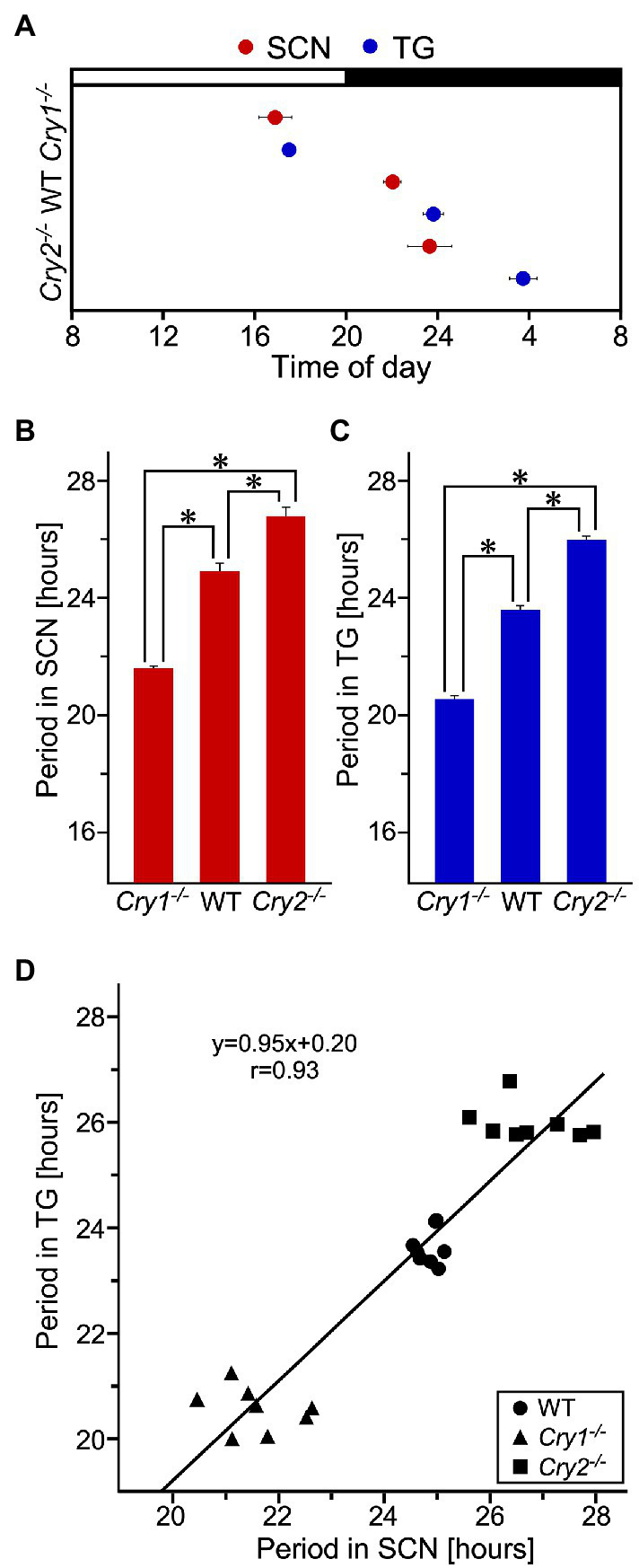
**Phase and period of circadian rhythms of PER2::LUC expression in the suprachiasmatic nucleus and the trigeminal ganglion of wild-type, *Cry1^−/−^* and *Cry2^−/−^* mice**. **(A)** Phase map for the suprachiasmatic nucleus (SCN) and the trigeminal ganglion (TG) of PER2::LUC mice. The average times (±SE) of the first peaks were plotted against the time of day (filled and empty bar indicates lighting times). **(B)** Circadian periods of PER2::LUC rhythm in the SCN of WT, *Cry1^−/−^*, and *Cry2^−/−^* mice. *p < 0.05 (Bonferroni’s test) **(C)** Circadian periods of PER2::LUC rhythm in the TG of WT, *Cry1^−/−^*, and *Cry2^−/−^* mice. *p < 0.05 (Bonferroni’s test) **(D)** Regression analysis of the data shown in **(B,C)**. The regression line (*r* = 0.93) is described by the formula *y* = 0.95*x* + 0.20, where *y* = period in TG and *x* = period in SCN (in hours).

PER2::LUC expression in the SCN and trigeminal ganglion showed strong, persistent circadian oscillations in the WT, *Cry1^−/−^*, and *Cry2^−/−^* mice (Figure 3). For the SCN and the trigeminal ganglion, the phase map of the first peaks was constructed (Figure 4A). In the WT, the phase of the first peak of the trigeminal ganglion was initiated significantly later than the phase of the SCN (trigeminal ganglion: 23.8 ± 0.5 h, SCN: 22.0 ± 0.4 h; *p* < 0.05, *t*-test). The first peak in *Cry 2^−/−^* mice was significantly later than the phase of the trigeminal ganglion compared to the SCN (trigeminal ganglion: 27.6 ± 0.6 h, SCN: 23.7 ± 1.0 h; *p* < 0.05, *t*-test). The phase of the first peak in *Cry1*^*−*/−^ mice was not significantly different between the trigeminal ganglion and the SCN (trigeminal ganglion: 17.5 ± 0.3 h, SCN: 16.9 ± 0.7 h; *p* = 0.44, *t*-test). In the SCN, *Cry1^−/−^* mice showed a significantly shorter circadian period than WT mice, while *Cry2^−/−^* mice showed a significantly longer period (Figure 4B; SCN of WT: 24.9 ± 0.3 h, that of *Cry1^−/−^*: 21.6 ± 0.1 h, that of *Cry2^−/−^*: 26.8 ± 0.2 h; *p* < 0.05, Bonferroni’s test, *n* = 8 per genotype). In addition, in the trigeminal ganglion, *Cry1^−/−^* mice had a significantly shorter circadian period than WT mice, while *Cry2^−/−^* mice had a significantly longer period (Figure 4C; trigeminal ganglion of WT: 23.6 ± 0.2 h, that of *Cry1^−/−^*: 20.5 ± 0.1 h, that of *Cry2^−/−^*: 26.0 ± 0.1 h; *p* < 0.05, Bonferroni’s test, *n* = 8 per genotype). The circadian periods in the trigeminal ganglion were shorter than those in the SCN for each genotype (*t*-test, *p* < 0.05 for all WT, *Cry1^−/−^*, and *Cry2^−/−^*). In addition, the circadian periods in the trigeminal ganglion of each genotype were correlated with the periods in the SCN (Single regression analysis, *r* = 0.93) (Figure 4D).

## 4. Discussion

In this study, we focused on the circadian rhythm of PER2 expression in the trigeminal ganglion and examined the role of the *Cry* gene in the regulation of the trigeminal ganglion’s circadian rhythm. Trigeminal ganglia *ex vivo* showed a circadian rhythm in PER2 expression. Immunohistochemistry showed that PER2 expression in the SCN and trigeminal ganglion was primarily localized in the neuronal cell bodies. Bioluminescence imaging also showed diurnal variation throughout the trigeminal ganglion tissue. These findings suggest that circadian oscillations at the tissue level in the trigeminal ganglion are generated primarily by signals from neurons.

The circadian period of the trigeminal ganglion was significantly shorter than that of the SCN in all genotypes and even showed circadian rhythms *ex vivo* independent of the SCN. Even when the trigeminal ganglion was removed and physically separated from the SCN, the circadian period of the trigeminal ganglion did not lose its rhythmicity. It continued with different rhythm than the SCN, indicating that it was not dependent on the expression of clock genes in the SCN. Thus, the trigeminal ganglion has a tissue-specific and autonomous circadian rhythm. However, *Per2* expression in the SCN always preceded that in the trigeminal ganglion in all genotypes, suggesting that the phase of the trigeminal ganglion may be regulated by some signals from the SCN, such as a hormonal or neural factor. The trigeminal ganglion has a different circadian period compared to the SCN, which may possibly be related to the effect of different neuron types and the cellular composition of each on clock gene expression in the same nerve. In all static cultures, the amplitude of the circadian rhythm was gradually dampened in all genotypes in both the SCN and the trigeminal ganglion, more so in the latter. In the trigeminal ganglion, the amplitude was significantly dampened after day 4. This may be caused by nutrient depletion as the medium in which they were cultured was not changed during the observation period. In the trigeminal ganglion, the cultured tissue and observed luminescence were about 10- and 5-fold larger than in the SCN, respectively, suggesting that nutrients were consumed more rapidly.

The first peak of the trigeminal ganglion was later than the first peak of the SCN in all genotypes. Previous reports have shown that the first peak of various peripheral tissues is later than that in the SCN at both the organism and tissue level. This relationship is no longer maintained when the SCN is destroyed (Yoo et al., 2004). In this study, the relationship between the SCN and trigeminal ganglion is preserved even in mice lacking a *Cry* gene, suggesting that the trigeminal ganglion is phase-adjusted by the SCN, a central clock, similar to other peripheral tissues. The function of the peripheral clock is to regulate rhythmic gene transcription of genes important to the physiological phenomena of each tissue, thereby tuning the physiological phenomena in the tissue to the optimal conditions of the environment. Therefore, the phase of the SCN rhythm is regulated by light information from the environment, and the trigeminal ganglion may receive the signal of the SCN rhythm as a peripheral clock. The circadian phase and period may be regulated so that sensory sensitivity in the trigeminal innervated area is the optimum condition for the environment.

*Cry1^−/−^* and *Cry2^−/−^* mice also showed circadian oscillations in PER2 expression in the SCN and the trigeminal ganglion. The circadian period was shorter in *Cry1^−/−^* mice and longer in *Cry2^−/−^* mice in both the SCN and trigeminal ganglion. The SCN periods observed in *Cry1^−/−^* or *Cry2^−/−^* mice were similar to those previously reported (Oda et al., 2022). The phase of the first peak in the trigeminal ganglion was also earlier in *Cry1^−/−^* and later in *Cry2^−/−^* mice than in WT mice. This change in phase was larger than the change in period. In other words, in the trigeminal ganglion, the absence of the *Cry* gene affected the period and phase of the first peak, suggesting that *Cry1* and *Cry2* regulate both the period and phase of the trigeminal ganglion. In the SCN, the amplitude of *Cry1^−/−^* decayed significantly in the fourth cycle; this was not observed in the *Cry2^−/−^* genotype. These results are consistent with previous reports suggesting that *Cry1* is required for the maintenance of circadian oscillations (Liu et al., 2007; Uchida et al., 2016).

There are several limitations to this study. This study focused on one aspect of the circadian rhythm of the trigeminal ganglion *in vivo*; it looked at the expression of clock genes in trigeminal ganglia taken from mice that were euthanized at a certain time of day. *In vivo*, diurnal variation in trigeminal- innervated areas has been reported for pain, but few studies have captured diurnal variation in areas such as tactile sensation in the dental and oral-facial regions. The diurnal variation of sensations other than pain in the trigeminal-innervated area and the direct relationship of each sensation to *Per*, *Cry*, and other clock genes will be the subject for future research. Also, our findings may apply to the trunk. This is because the trigeminal ganglion, which transmits somatosensory perception in the maxillofacial region, and the spinal dorsal root ganglion, which transmits somatosensory perception in the trunk, are considered anatomically and functionally equivalent. There have actually been reports of circadian rhythms in clock gene expression in the spinal dorsal root ganglion (Kim et al., 2020), but the diurnal variation in sensory perception in peripheral tissues will need to be further investigated. In addition, the mice were allowed to consume food and water *ad libitum* in this study; their feeding behavior was in accordance with the circadian rhythm of the mice’s life cycle (Marchant and Mistlberger, 1997). In the trigeminal ganglion, which is receptive to sensations in the teeth and oral region, the circadian rhythm may have been affected by the rhythm of tactile and pressure sensations caused by feeding behavior. Further research is needed to understand the diurnal variation of sensory perception at the cellular and tissue level and the rhythm of clock gene expression when stimulation of the trigeminal nerve is altered by tooth extraction or restricted feeding.

The trigeminal ganglion is a collection of cell bodies of the trigeminal nerve that transmits sensory information in the dental and oral regions. In this study, we show for the first time that an autonomous circadian rhythm exists in the trigeminal ganglion. The trigeminal nerve-innervated area is where glossodynia (a tingling pain in the tongue) and hypersensitivity manifest even in the absence of an organic problem. In the past, we reported that pain, one of the senses in the trigeminal-innervated area, has diurnal variation and that mice exhibited increased pain in the dark period (Niiro et al., 2021). These results may provide further evidence for this. Thus, the present study suggests that the sensations transmitted by the trigeminal ganglion may have diurnal variation. It may be due to circadian rhythms of the whole organism that depend on signals from the SCN, and on circadian rhythms of clock genes (such as *Per2*) in the trigeminal ganglion itself. In medical research targeting clock genes, CRY1 may be related to the development of bladder cancer and leukemia (Hanoun et al., 2012; Jia et al., 2021), and therapeutic agents selective for CRY2 have been reported to be effective in treating glioblastoma, a malignant brain tumor (Miller et al., 2022). As such, future development of new treatment methods will likely focus on the relationship between various diseases of the trigeminal nervous system and clock genes.

## Data availability statement

The original contributions presented in the study are included in the article/Supplementary material, further inquiries can be directed to the corresponding authors.

## Ethics statement

The animal study was reviewed and approved by the Animal Care and Use Committee of Kagoshima University and the Animal Care and Use Committee at Nagasaki University.

## Author contributions

YS, SNO, and WN designed the study. YS, SNO, KAY, and YO performed the experiment. YS, SNO, EK, YO, TJN, WN, and MS analyzed the data. YS, TJN, WN, and MS wrote the manuscript. All authors contributed to the article and approved the submitted version.

## Funding

This study was supported by a Grants-in-Aid from the Japan Society for the Promotion of Science (JSPS) for Scientific Research (19K10336 to SNO, 20K10099 to MS). This study was also supported by a Grants-in-Aid from the Ministry of Education, Culture, Sports, Science and Technology (MEXT) for Scientific Research (JP19K10058, JP22K09916 to EK), and for Scientific Research on Innovative Areas (“Brain Information Dynamics,” JP22H05162 to EK).

## Conflict of interest

The authors declare that the research was conducted in the absence of any commercial or financial relationships that could be construed as a potential conflict of interest.

## Publisher’s note

All claims expressed in this article are solely those of the authors and do not necessarily represent those of their affiliated organizations, or those of the publisher, the editors and the reviewers. Any product that may be evaluated in this article, or claim that may be made by its manufacturer, is not guaranteed or endorsed by the publisher.
